# Treatment of a Carpal Giant Cell Tumor of Bone With Curettage and Cemented Capitohamate Fusion

**DOI:** 10.1016/j.jhsg.2024.05.004

**Published:** 2024-06-14

**Authors:** Seth Ahlquist, Jordan S. Gross, Scott D. Nelson, Nicholas M. Bernthal, Lauren E. Wessel

**Affiliations:** ∗ Department of Orthopaedic Surgery, David Geffen School of Medicine at UCLA, Los Angeles, CA

**Keywords:** Capitate, Carpal fusion, Cement, Giant cell tumor of bone, Hamate

## Abstract

Carpal giant cell tumor of bone spanning multiple bones is a rare condition. We present a case of a man in his fifth decade with wrist pain who was found to have giant cell tumor of bone involving his capitate and hamate bones. This condition was successfully treated with intralesional curettage, argon beam coagulation, chemical cauterization and a cemented limited carpal fusion with satisfactory outcomes and no recurrence at 1-year postoperative follow-up.

Giant cell tumor of bone (GCT) accounts for approximately 5% of primary bone tumors.^1^ It is uncommon in the hand, accounting for only 2% to 5% of all tumors, and is even more uncommonly found in the carpals.^2^ When located within the carpus, GCT typically occurs as a single lesion, with the involvement of multiple bones being exceedingly rare.^3^, ^4^, ^5^, ^6^ There is a single report in the literature of a concurrent capitate and hamate GCT.^3^ While GCT is benign, it is locally aggressive with a high rate of recurrence if soft tissue extension exists, especially when treated with intralesional curettage in isolation.^1^^,^^7^ In general, treatment strategies have sought to avoid recurrence with en bloc resection and adjunctive procedures for margin expansion such as bone grafting, argon beam coagulation, chemical cauterization with peroxide or phenol, and cementation, with the aim of preserving wrist motion in order to optimize long term functional outcomes.^1^, ^2^, ^3^, ^4^, ^5^, ^6^^,^^8^, ^9^, ^10^

The rarity of carpal GCTs and the further rarity of those that span multiple small bones makes optimal treatment uncertain. Whether these need to be treated as Campanacci grading suggests, given the spanning of joint and soft tissues, for risk of recurrence is yet unknown. The purpose of this article is to describe a surgical technique for the treatment of a carpal giant cell tumor of bone that spanned multiple carpals with curettage, cementation, and limited carpal fusion.

## Case Report

A 53-year-old right-handed man with a prior history of a right parietal lobe oligoastrocytoma status post resection, chemotherapy and radiation therapy 14 years prior, with residual left-sided weakness, presented to orthopedic oncology clinic for 2 months of atraumatic right wrist pain. Pain was worse with activity and had no associated neurovascular symptoms. On examination, the patient exhibited painful range of motion (ROM) of the wrist (30° flexion and 30° extension), and dorsal carpal tenderness with deep palpation.

**Figure 1:**
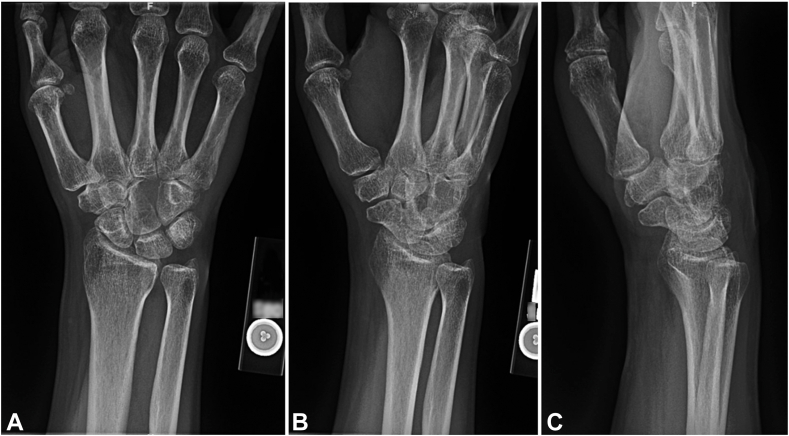
**A–C** Plain radiographs of the wrist demonstrating destructive osteolytic lesion of the capitate and hamate.

**Figure 2:**
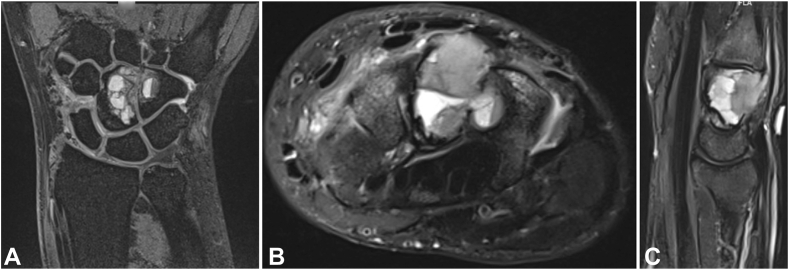
**A–C** Magnetic resonance imaging coronal and axial T2-weighted and sagittal inversion recovery sections showing a hypointense expansile, destructive lesion originating in the capitate with extension into the hamate.

**Figure 3:**
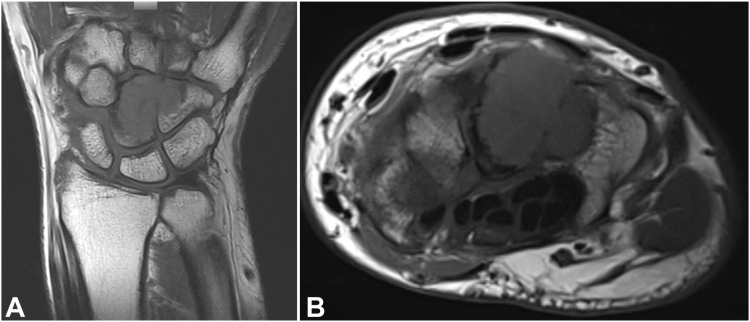
**A, B** Magnetic resonance imaging T1-weighted coronal and axial sections showing an isointense expansile, destructive lesion originating in the capitate with extension into the hamate.

**Figure 4:**
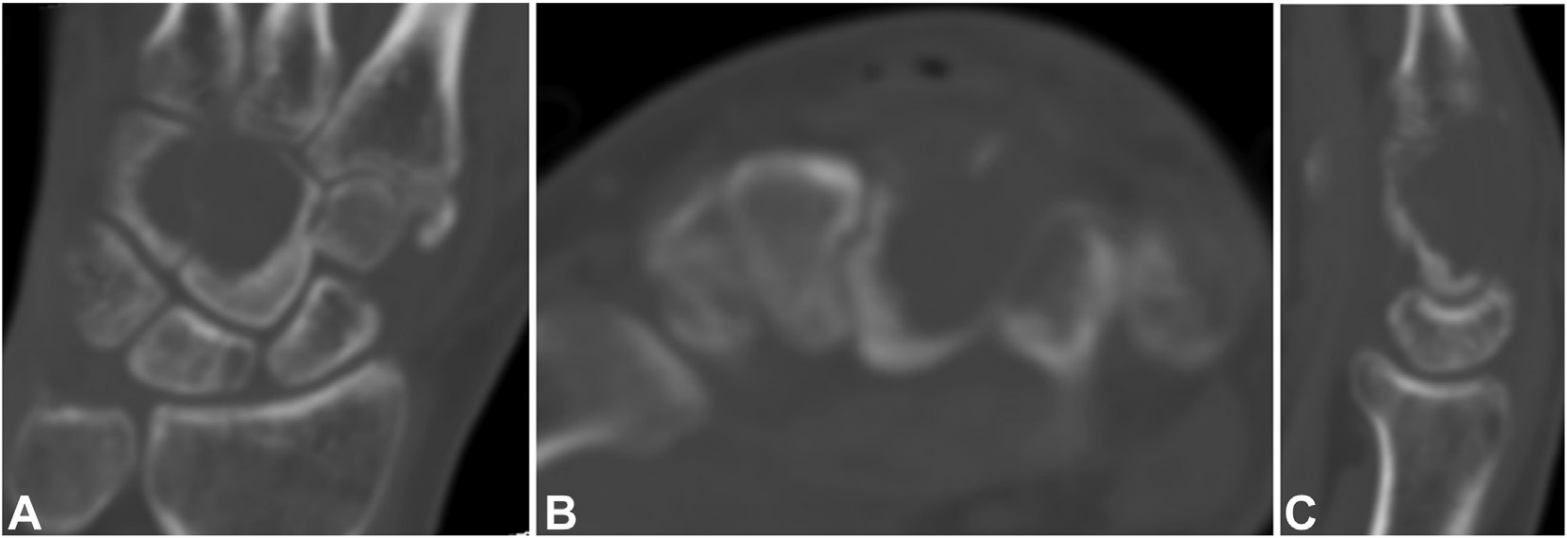
**A–C** Computed tomography imaging coronal, axial and sagittal sections showing a destructive osteolytic lesion of the capitate and hamate.

**Figure 5:**
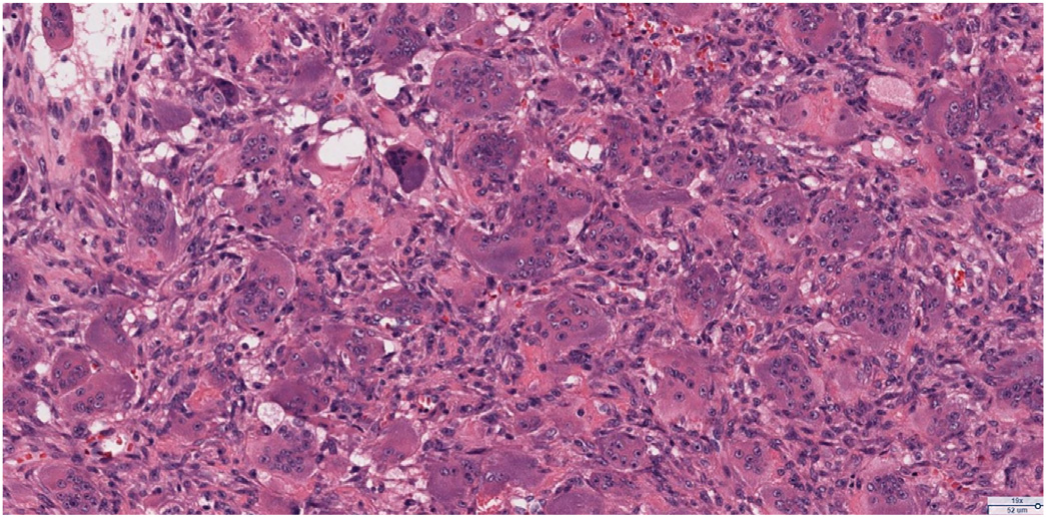
High-powered histological section photomicrograph demonstrating a giant cell-rich neoplasm with abundant giant cells containing numerous nuclei with intervening mononuclear cells demonstrating nuclear characteristics similar to the multinucleated giant cells (Hematoxylin-eosin stain; magnificiation × 160).

Standard radiographs demonstrated an expansile lytic lesion involving the capitate as well as part of the hamate with cortical destruction, Campanacci Grade III (Fig. 1). Magnetic resonance imaging of the wrist demonstrated a T1 isointense and T2 hypointense expansile lesion originating in the capitate with violation of the dorsal and ulnar cortex and extension into the hamate with multiple fluid-fluid levels and post-contrast enhancement (Figure 2, Figure 3). The patient underwent a CT-guided bone biopsy to confirm the diagnosis (Fig. 4). Histopathology revealed a giant cell-rich neoplasm with abundant giant cells containing numerous nuclei with intervening multinucleated giant cells (Fig. 5). Treatment options were discussed, and the patient elected to proceed with operative management. Written informed consent was obtained from the patient for publication of this case report and accompanying images. The authors adhered to CARE (for CAse REports) guidelines for writing this case report.

### Surgical technique

A dorsal approach to the wrist was performed and an incision was made in line with the third metacarpal. Extensor pollicis longus tendon was identified and protected. An incision was made between the second and fourth extensor compartments to expose the dorsal capsule, where notable ulnar carpal soft tissue swelling was noted. A ligament splitting approach was used to expose the tumor below. Gross tumor was found between the capitate and hamate extending into the dorsal soft tissues, measuring 4 × 3 × 0.5 cm and comprised of tan-red irregular soft tissue fragments. The most distal aspect of the capsulotomy was extended to provide additional visualization for adequate curettage.

**Figure 6:**
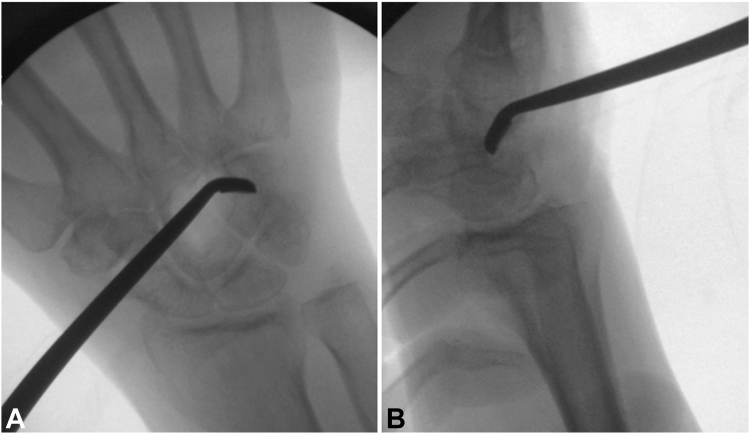
**A, B** Intraoperative fluoroscopic images demonstrating intralesional currettage.

The tumor was subsequently curettage, leaving a void (Fig. 6). A bony hole was noted between the ulnar surface of the capitate and the radial surface of the hamate. The void was irrigated with peroxide, and a high-speed bur and argon beam were used for margin expansion.

### Fixation/reconstruction

**Figure 7:**
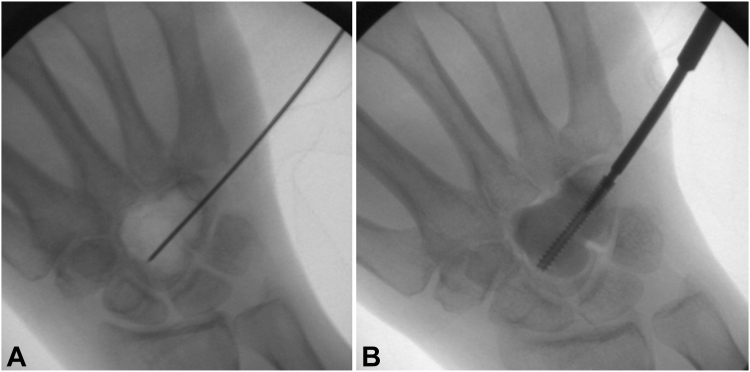
**A, B** Intraoperative fluoroscopic images demonstrating guidewire and headless compression screw placement.

**Figure 8:**
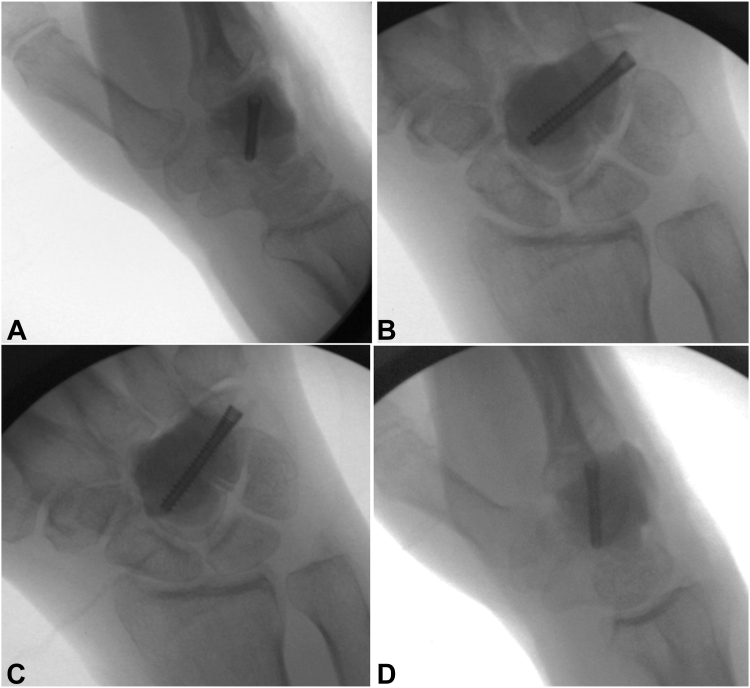
**A–D** Intraoperative fluoroscopic images demonstrating cementation around the capitohamate fusion with a headless compression screw.

**Figure 9:**
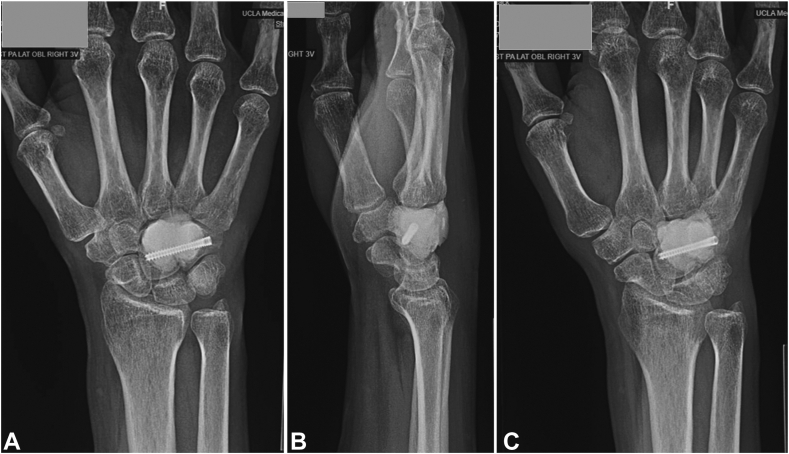
Postoperative plain radiographs status post cemented capitohamate fusion.

Under fluoroscopic visualization, a 0.045 inch guide wire was started at the ulnar aspect of the hamate and driven radially and proximally across the capitate (Fig. 7A). The start point was drilled and a 26 mm Acutrak 2 mini headless compression screw (Acumed Inc) was partially advanced (Fig. 7B). Palacos bone cement (ZimmerBiomet) was mixed, allowed to partially harden, and then inserted into the void (Fig. 8A, B). While the cement was still soft, the Acutrak screw was driven across the tumor void for a rebar-type fixation (Fig. 8C, D). Appropriate positioning and fill of cement were confirmed on fluoroscopy and postoperative radiographs (Fig. 9). The tourniquet was let down, and hemostasis was obtained.

The dorsal capsulotomy was closed with #0 Vicryl suture (Ethicon). 2-0 Vicryl sutures were used to close the subcutaneous tissue and skin was closed with 4-0 nylon sutures. The patient was placed in a sterile bulky dressing and a volar forearm splint in functional hand position.

### Rehabilitation

**Figure 10:**
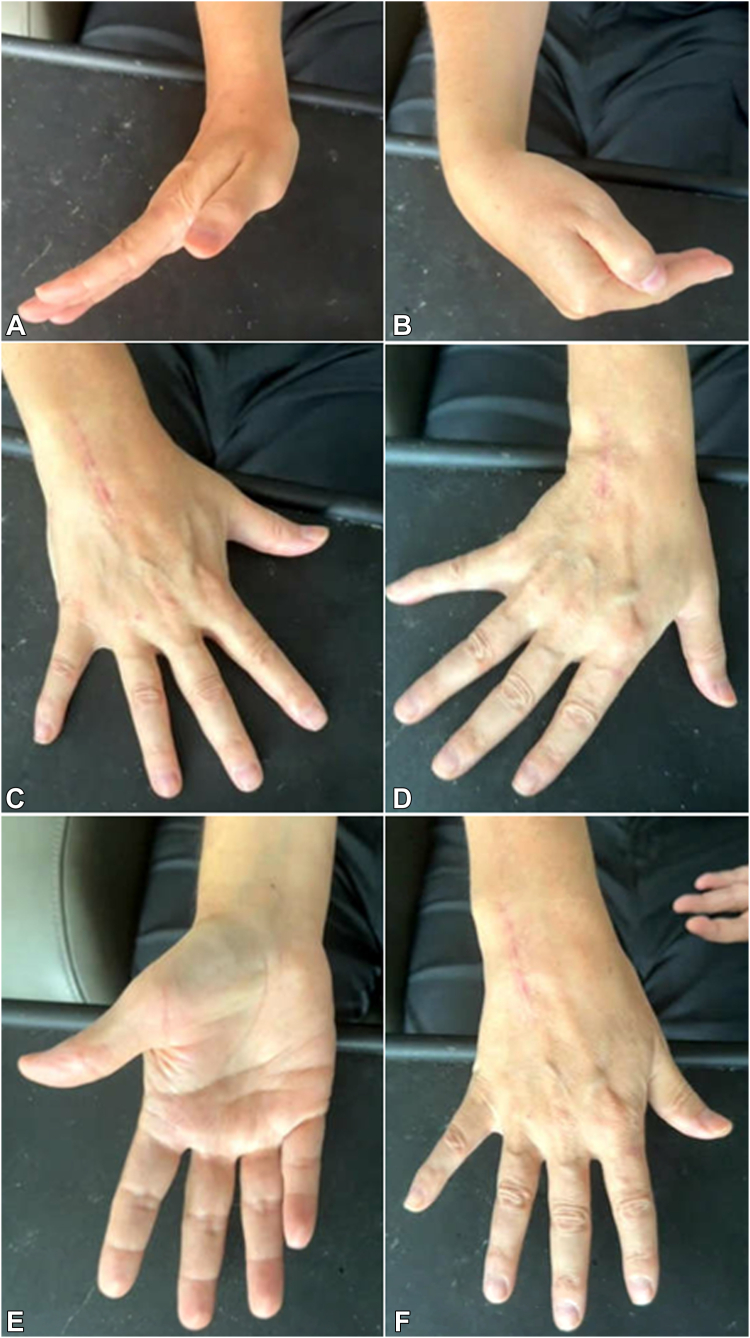
**A–F**. Postoperative clinical photos, 15 months status post cemented capitohamate fusion. **A, B** Wrist flexion/extension; **C, D** wrist radioulnar deviation; **E, F** wrist pronosupination.

**Figure 11:**
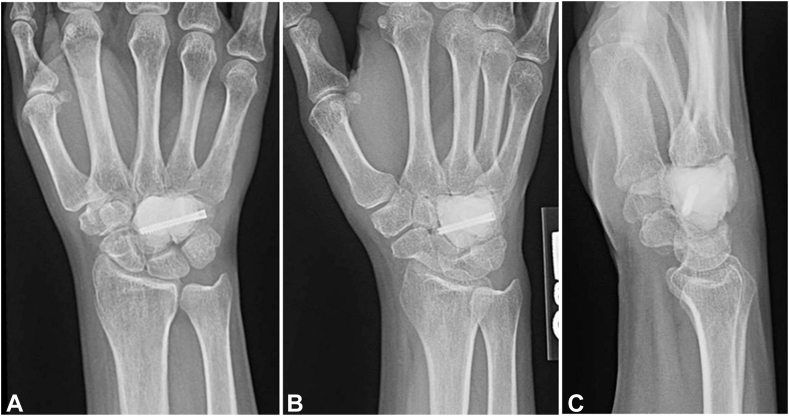
**A–C** Plain radiographs 15 months after surgery from cemented capitohamate fusion.

At 2 week follow-up, the sutures and splint were removed, and the patient was transitioned to a removable wrist brace with continued nonweightbearing. At the 6 weeks postsurgery time point, the incision was healed, the removable wrist brace was discontinued, and the patient began progressive weight bearing. At 15 months after surgery, the patient had not been working on ROM, nor started formal hand therapy because of a recent ventriculoperitoneal shunt placement for normal pressure hydrocephalus. At this time, the patient had wrist ROM of 45° extension, 50° flexion, full pronosupination, 15° radioulnar deviation, with good grip and pinch strength similar to the contralateral side, and was able to make a composite fist (Fig. 10A**–**F). The patient reported minimal pain and some mild wrist stiffness but felt that overall his hand/wrist was functional for daily activities. Radiographs at this time demonstrated no gross evidence of tumor recurrence, and the implant was in stable position (Fig. 11). Chest imaging remained negative for pulmonary metastasis.

## Discussion

Carpal GCT is exceedingly rare but nevertheless remains a challenging management problem.^1^^,^^2^ Prior case reports and literature reviews have demonstrated 10 instances of unifocal capitate 7 reports of unifocal hamate, and 4 instances of carpal GCTs spanning multiple bones, of which only one had isolated capitohamate involvement.^3^, ^4^, ^5^, ^6^^,^^8^, ^9^, ^10^ Gupta et al^5^ reported an instance of capitate, hamate, and triquetrium involvement, which was treated with distal row carpectomy, iliac autograft reconstruction, and fusion of the distal carpal segment to the metacarpals with Kirschner wires, finding no recurrence and a return to preoperative hand function at 18 months (however, no specific ROM data was reported). In a skeletally immature patient, Ansari et al^6^ reported an instance of capitate, hamate, trapezoid, and third metacarpal GCT that was treated with en bloc resection, fibular allograft reconstruction, and proximal row to metacarpal fusion with locking plates, resulting in no recurrence at 22 months after surgery and functional wrist motion (40° extension, 30° flexion, and 10° radioulnar deviation). Two additional case reports also performed en bloc resection with the same fusion technique for capitohamate and capitate GCT and found no recurrence with reasonable wrist function at 1 and 2 years after surgery (40 and 30° extension, 35 and 25° wrist flexion, respectively).^3^^,^^9^ Tarng et al^4^ reported an instance of scaphoid, capitate, trapezium, and trapezoid involvement which was treated with en bloc resection, iliac autograft reconstruction and fixation with Kirschner wires/mini screws, with no recurrence at 1 year and wrist ROM of 40° extension and 50° flexion. All of these prior reports employed some form of en bloc resection, bone graft reconstruction, and carpal to metacarpal fusion, resulting in functional wrist motion ranging from 30-40° extension, 30–50° of flexion, 10–15° radioulnar deviation, and 50% to 90% grip strength at 1–2 years follow-up.^3^^**–**^^6^^,^^9^

In the present study, a more limited carpal fusion was performed involving only the distal carpal row between the capitate and hamate, which likely allowed our patient to have improved ROM and hand/wrist function compared with the other patients in these studies. This is especially notable given that our patient achieved this function despite minimal to no participation in hand therapy or home exercises since an even greater postoperative ROM would be expected if he were to have participated in therapy. Prior analyses have shown that there is a noninsignificant amount of flexion/extension between the capitate and lunate, and therefore, it is likely ideal to avoid fusing across the midcarpal joint when possible in order to preserve postoperative ROM. Capitohamate fusion, as employed in the present study, is an uncommon partial wrist fusion but has been previously described as a treatment for end stage Kienböck disease.^11^ We also elected for cement to fill the void after surgical excision given the ability to obtain additional margin expansion and the ability to detect recurrence, which is a well-described treatment strategy for GCT but has also been previously reported in the treatment of unifocal capitate and hamate GCT.^8^^,^^9^

Herein, we describe a novel technique for the rare condition of a carpal GCT. Our strategy involved an intralesional curettage with electrocoagulation, chemical cauterization, and a limited midcarpal fusion. Our report suggests that en bloc resection may not be required for GCTs that span multiple small bones and an intralesional procedure with limited carpal fusion may allow for decreased functional morbidity without increasing the rate of local recurrence. However, longer term follow-up is required to confirm the durability of this treatment strategy.

## Conflicts of Interest

No benefits in any form have been received or will be received related directly to this article.
